# Short-term study fails to capture negative impacts of livestock intensification on wildlife

**DOI:** 10.1073/pnas.2502418122

**Published:** 2025-05-30

**Authors:** Joseph O. Ogutu, Jared A. Stabach, J. Grant C. Hopcraft, Randall B. Boone, Holly T. Dublin, Christopher L. Dutton, Andrew Gichira, Abby Guthmann, Ask L. Herrik, Kay E. Holekamp, Rebekah R. Karimi, Shem C. Kifugo, Peter Leimgruber, Niels Mogensen, Stephen S. Moiko, Joseph M. Mukeka, Harrison Nabaala, Stephen Ndambuki, Lucy M. Njino, Gordon O. Ojwang, Han Olff, Craig Packer, Lemein Parmuntoro, Robin S. Reid, Rehema B. Rioba, Mohammed Y. Said, Jully S. Senteu, Amanda L. Subalusky, Jens-Christian Svenning, Stewart Thompson, Antonio Uzal, Michiel P. Veldhuis, Robert Buitenwerf

**Affiliations:** ^a^Biostatistics Unit, Institute of Crop Science, University of Hohenheim, Stuttgart 70599, Germany; ^b^Conservation Ecology Center, Smithsonian’s National Zoo and Conservation Biology Institute, Front Royal, VA 22630; ^c^School of Biodiversity, One Health and Veterinary Medicine, University of Glasgow, Glasgow G12 8QQ, United Kingdom; ^d^Natural Resource Ecology Laboratory, Department of Ecosystem Science and Sustainability, Colorado State University, Fort Collins, CO 80521; ^e^International Union for Conservation of Nature (IUCN) Eastern and Southern Africa Regional Office, Nairobi 00200, Kenya; ^f^Department of Biology, University of Florida, Gainesville, FL 32611-8525; ^g^Centre for Ecosystem Restoration, Nairobi 00217, Kenya; ^h^Department of Ecology, Evolution, and Behavior, University of Minnesota, St. Paul, MN 55108; ^i^Department of Biology, Center for Ecological Dynamics in a Novel Biosphere, Aarhus University, Aarhus DK-8000, Denmark; ^j^Department of Integrative Biology, Michigan State University, East Lansing, MI 48824-1115; ^k^Kenya Bird of Prey Trust, Naivasha 20117, Kenya; ^l^Groningen Institute for Evolutionary Life Sciences, University of Groningen, Groningen, AG 79747, The Netherlands; ^m^Kenya Wildlife Trust, Nairobi 00502, Kenya; ^n^Nabara Consult, Nairobi 00206, Kenya; ^o^Wildlife Research and Training Institute, Naivasha 842-20117, Kenya; ^p^Directorate of Resource Surveys and Remote sensing, Nairobi 00100, Kenya; ^q^Department of Ecosystem Science and Sustainability, Colorado State University, Fort Collins, CO 80523; ^r^College of Biological and Physical Sciences, Institute for Climate Change and Adaptation, University of Nairobi, Nairobi 00100, Kenya; ^s^School of Animal, Rural and Environmental Sciences, Nottingham Trent University, Southwell NG25 0QF, United Kingdom; ^t^Department of Environmental Biology, Institute of Environmental Sciences, Leiden University, Leiden 2333CC, The Netherlands

Xu and Butt (XB; 1) claim that livestock grazing does not affect wild herbivores in Kenya’s Maasai Mara National Reserve, downplaying widespread wildlife declines caused by intensifying land use and livestock grazing (2, 3) because their study spanned an anomalously wet 19-mo period when livestock–wildlife competition was likely the lowest in 50 y (4). They fail to recognize the Reserve’s critical role as a last refuge for the region’s wildlife, especially during recurrent droughts, and ignore impacts on locally rare wild herbivores and large carnivores. Further, their study suffers irredeemable methodological flaws: It relies on 440 cattle dung piles from a 3-hectare area, ignores spatiotemporal dynamics of wild herbivore abundance and distribution, and unjustifiably models environmental seasonality with a smooth, symmetric function. Consequently, their conclusions are inadequate to support their recommendation to allow livestock grazing in the Reserve.

**Fig. 1.**
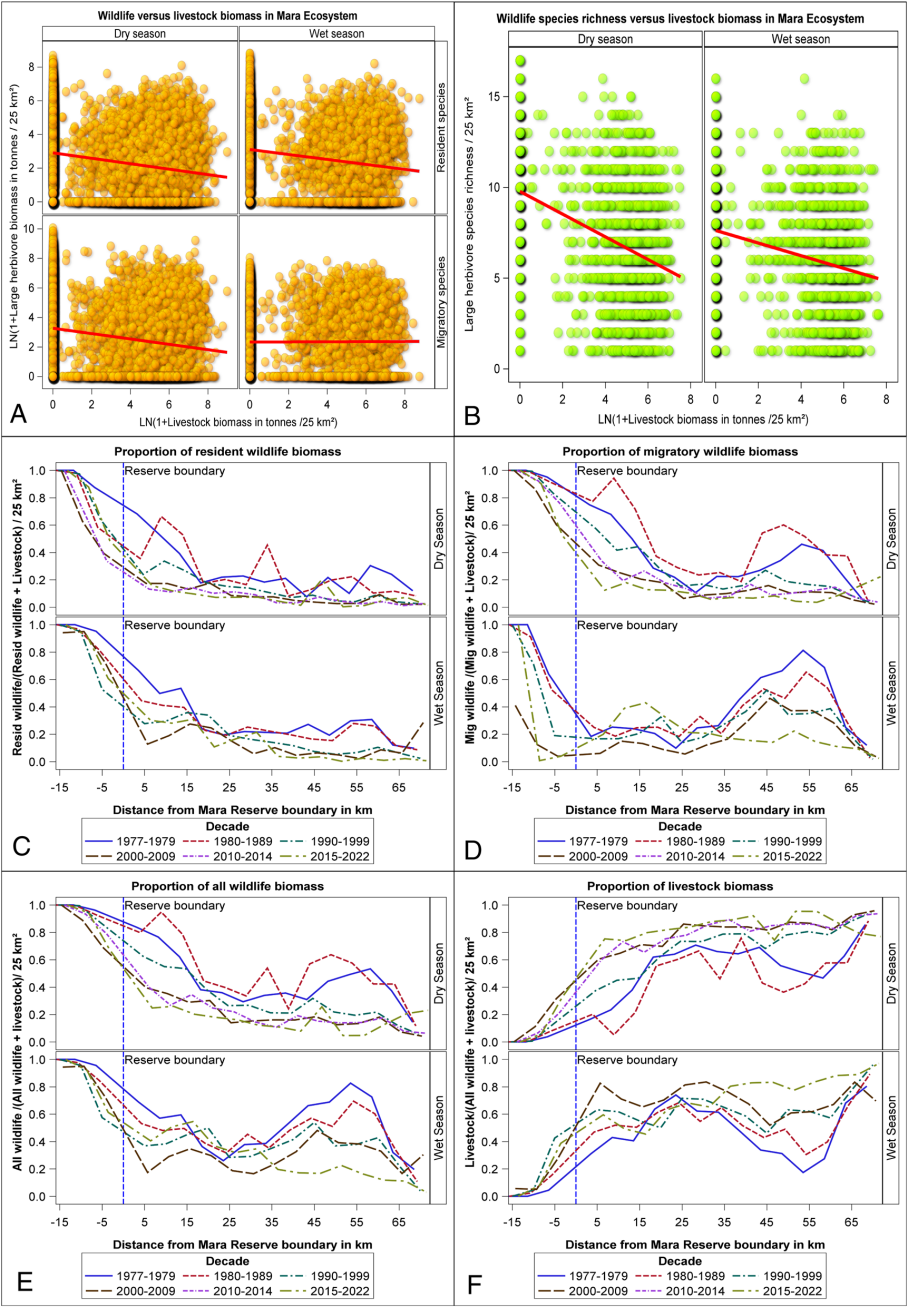
Logarithm of aggregate large herbivore (≥15 kg) biomass (tons/25 km^2^) as a function of the logarithm of livestock (cattle, donkeys, sheep, and goats) biomass (tons/25 km^2^) (*A*) and large herbivore species richness/25 km^2^, averaged over each decade (*B*) across the 7,500 km^2^ Masai Mara Ecosystem based on 77 systematic reconnaissance aerial surveys conducted by Kenya’s DRSRS between 1977 and 2022 and one aerial total count conducted jointly by the Kenya Wildlife Research and Training Institute and the Kenya Wildlife Service in 2021. In the dry season, biomass of resident (*r*_s_ = −0.23589, *P* < 0.0001, n = 11,838) and migratory (*r*_s_ = −0.21419, *P* < 0.0001, n = 11,838) wildlife—including wildebeest, zebra, Thomson’s gazelle, and eland—decreases significantly with increasing livestock biomass. In the wet season, resident wildlife biomass (*r*_s_ = −0.22782, *P* < 0.0001, n = 7,736) also decreases significantly with increasing livestock biomass but migratory wildlife (*r*_s_ = −0.00264, *P* < 0.8166, n = 7,736) does not. Species richness decreases significantly with increasing livestock biomass density in both the dry (*r*_s_ = −0.30992, *P* < 0.0001, n = 1,591) and wet (*r*_s_ = −0.25015, *P* < 0.0001, n = 1,548) seasons. The solid red lines show the fitted linear regression lines. The proportions of resident (*C*), migratory (*D*), and overall (*E*) wildlife biomass increase toward and inside the Mara reserve, whereas the proportion of livestock biomass (*F*) decreases, indicating that livestock activity compresses wildlife into the reserve boundaries, reducing their presence in surrounding areas.

**Fig. 2.**
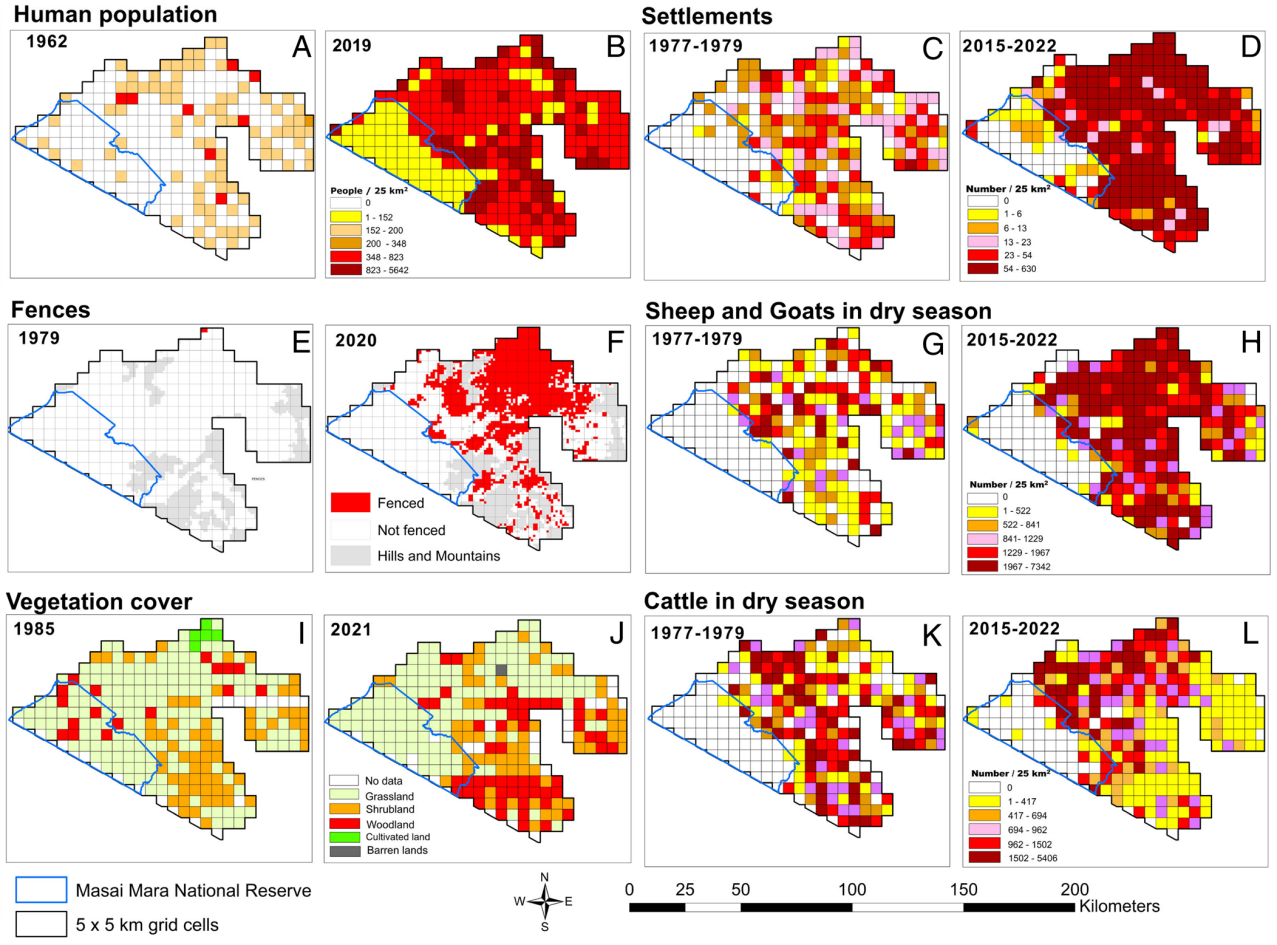
Intensifying pressures throughout the Masai Mara Ecosystem are evident in multiple trends: rapidly increasing human population between 1962 and 2019 (*A* and *B*); the expansion of settlements between 1970s and 2015–2022 (*C* and *D*); the proliferation of fences between 1985 and 2020 (*E* and *F*); growing sheep and goat numbers between 1970s and 2015–2022 (*G* and *H*); increasing bush encroachment between 1985 and 2021 (*I* and *J*); and increasing cattle density and spatial spread between 1970s and 2015–2022 (*K* and *L*).

Since 1977, Kenya’s Directorate of Resource Surveys and Remote Sensing (DRSRS) has conducted 77 ecosystem-wide aerial surveys, revealing over 70% declines in all wild herbivores >15 kg except elephants, alongside a 269% increase in sheep and goats and a 13% decrease in cattle (2, 3). These declines threaten the long-term viability of wildlife populations and have resulted in the local extinction of roan antelope, beisa oryx, and wild dog. Moreover, wildebeest, zebra, Thomson’s gazelle, and eland migrations between the Mara-Loita Plains collapsed during 2015–2020, largely due to livestock-related fencing (5). Although livestock are prohibited in the Reserve, cattle tracks radiating from Talek are visible from space (2, 6), with livestock grazing increasing >500% Reserve-wide since 1977, intensifying negative impacts on wild herbivore biomass and diversity (Fig. 1; 2, 3).

XB ignore empirical evidence on how human land-use intensification affects the region’s biodiversity (6), fail to recognize wildlife avoidance of areas with heavy livestock grazing during drought (7), and disregard retaliatory predator killings (8) and knock-on effects on ecosystem processes, including fire (2), that are all strongly linked to livestock. While we acknowledge that livestock grazing can promote forage quality and facilitate smaller-bodied herbivores, evidence shows that larger herbivores (e.g., buffalo, elephant) avoid cattle, contradicting XB’s conclusions (9). Given the importance and ongoing dramatic declines of megafauna in Kenya (3) and globally, allowing livestock into the increasingly pressured Reserve is indefensible.

We agree with XB that pastoralists are long-standing stewards of the Mara ecosystem and play a vital role in addressing the complex challenges regarding the region’s conservation. Characterizing protected areas with minimal human impact as a “fetishism of pristine wilderness,” however, ignores both their well-documented ecological importance and critical role in maintaining biodiversity (10). Protected areas cover a mere 8% of Kenya’s land, providing some of the last remaining refuges where significant wildlife populations still roam, despite increasing anthropogenic pressures (Fig. 2; 2, 10). In 2023/24, the Reserve generated >$33.5 million in fees, 91% of Narok County’s total revenue, primarily from tourists drawn to experience the region’s extraordinary wildlife. Encouraging livestock grazing in the Reserve would almost certainly exacerbate already strong livestock-related impacts on wildlife, accelerate wildlife declines, jeopardize ecotourism, and have far-reaching negative ecological and economic consequences.
